# Therapeutic potential of small extracellular vesicles derived from mesenchymal stem cells for spinal cord and nerve injury

**DOI:** 10.3389/fcell.2023.1151357

**Published:** 2023-03-22

**Authors:** Young-Ju Lim, Gyeong Na Jung, Wook-Tae Park, Min-Soo Seo, Gun Woo Lee

**Affiliations:** ^1^ Department of Orthopedic Surgery, Yeungnam University College of Medicine, Yeungnam University Medical Center, Daegu, Republic of Korea; ^2^ Department of Veterinary Tissue Engineering, College of Veterinary Medicine, Kyungpook National University, Daegu, Republic of Korea

**Keywords:** nerve, spinal cord, mesenchymal stem cell (MSC), extracellular vesicles (EV), small EV

## Abstract

Neural diseases such as compressive, congenital, and traumatic injuries have diverse consequences, from benign mild sequelae to severe life-threatening conditions with associated losses of motor, sensory, and autonomic functions. Several approaches have been adopted to control neuroinflammatory cascades. Traditionally, mesenchymal stem cells (MSCs) have been regarded as therapeutic agents, as they possess growth factors and cytokines with potential anti-inflammatory and regenerative effects. However, several animal model studies have reported conflicting outcomes, and therefore, the role of MSCs as a regenerative source for the treatment of neural pathologies remains debatable. In addition, issues such as heterogeneity and ethical issues limited their use as therapeutic agents. To overcome the obstacles associated with the use of traditional agents, we explored the therapeutic potentials of extracellular vesicles (EVs), which contain nucleic acids, functional proteins, and bioactive lipids, and play crucial roles in immune response regulation, inflammation reduction, and cell-to-cell communication. EVs may surpass MSCs in size issue, immunogenicity, and response to the host environment. However, a comprehensive review is required on the therapeutic potential of EVs for the treatment of neural pathologies. In this review, we discuss the action mechanism of EVs, their potential for treating neural pathologies, and future perspectives regarding their clinical applications.

## 1 Introduction

Neural structural pathologies, such as traumatic injury and demyelinating disease, are uncommon conditions, and strategies for effective management are lacking (Burnett and Zager, 2004; Menorca et al., 2013; Tyler et al., 2013; Wang and Pearse, 2015; Morales et al., 2016). Several experimental studies have demonstrated that critical processes occur after neuronal damage (Carlson and Gorden, 2002; Kim et al., 2021a). In cases of traumatic neural damage, secondary injury of neural structures within several minutes of primary injury causes hemorrhage, swelling, ischemia, disrupts feeding vessels, and damages neural tissues (Aiyer et al., 2021), and pro-inflammatory cytokines and chemokines, such as interleukin-1 and 6 (IL-1 and -6) and tumor necrosis factor-alpha (TNF-α), play significant roles in the progression and aggravation of neural structure damage in most processes associated with neural injury (Qian et al., 2022).

Despite recent improvements in the management of neural pathologies, treatments are limited. Therapeutic strategies can be divided into three broad categories; surgical decompression, anti-inflammatory treatment, and axonal regeneration. Surgical decompression involves the removal of causes of mechanical compression in cases of traumatic damage (Rath and Balain, 2017; Wutte et al., 2019). Anti-inflammatory treatments are administered in regions surrounding injured nerves (such as in cases of spinal cord injury) to suppress inflammatory mechanisms and factors associated with spinal cord injury during the early and chronic phases. Axonal regeneration at sites of neural injury is the ultimate treatment goal. During the early phase of neural injury, macrophages infiltrate lesions and form wound cavities around lesion sites that induce scarring and the formation of fluid cavitations that prevent neuronal regeneration, as axons cannot bridge the liquid contents of these cavities. In addition, arachnoiditis, a granulomatous infiltration around damaged nerves, contributes to the formation of mature scars that do not contain astrocytes or glial cells (Seki and Fehlings, 2008; Vismara et al., 2017; Bhat et al., 2019; Liau et al., 2020).

Cell-based therapies, such as stem cell treatments, have been investigated as means of regenerating neural damage by replacing damaged cells and creating cellular environments conducive with healing (Chen et al., 2021; Sykova et al., 2021). Mesenchymal stem cells (MSCs) suppress inflammation and limit secondary injury, secrete paracrine factors that protect remaining axons and promote axonal regeneration, and differentiate into nerve cells that replace damaged nerve cells. In addition, the synthesis of neurotrophic and angiogenic factors by MSCs promotes their differentiation into neuron-like cells and neuronal survival. Furthermore, MSC treatments are safe, and the immunomodulatory properties of these cells make them therapeutically valuable (Huang et al., 2021). Although MSCs can regenerate injured tissues and control immunologic cascades, they also have several limitations. First, the survival of MSCs after implantation would be limited, as their longevities may be influenced by the cellular environment and intercellular communication. In particular, it was reported that after MSC implantation, pro-inflammatory activity might temporarily surpass anti-inflammatory activity (Kretlow et al., 2008) and that this adversely affects MSC function and survival. Second, MSCs are heterogeneous due to donor-associated differences, cell type, and differentiation capacity, and extremely sensitive to environmental factors, which negatively affect the ability of MSCs to, for example, attenuate severe inflammation and active osteoarthritis. Third, the method used to manufacture MSCs, including *ex vivo* expansion, isolation techniques, and cultivation methods, has not been standardized, and undetermined factors can induce MSC senescence and loss of function.

Extracellular vesicles (EVs) have recently emerged as a novel alternative in regenerative and anti-inflammatory medicine (Witwer et al., 2021; Sung et al., 2022a). This review introduces EVs as novel therapeutic source for regenerative medicine and includes details of the isolation and characterization of small EVs, the relationship between MSCs and small EVs, and a summary of experimental evidence supporting the therapeutic use of EVs for the treatment of neural injuries. In addition, we discuss future prospects and developmental challenges and directions.

## 2 What are EVs and small EVs?

**FIGURE 1:**
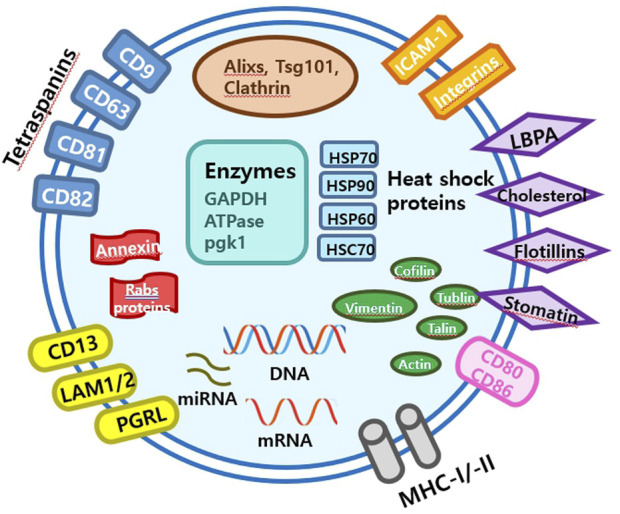
Schematic diagram of an small EVs.

Extracellular vesicles are lipid-bound vesicles secreted into the extracellular space. There are three main types of EVs; microvesicles, small EVs, and apoptotic bodies (Figure 1), which contain lipids, nucleic acids, and proteins, particularly those associated with cytoplasm and metabolism. No specific protein markers have been identified that can differentiate the three EV types (Kim et al., 2021a). Although their protein profiles differ, they overlap, possibly because of a lack of isolation and analytical methods. Notably, extracellular vesicles may be used as couriers of cell-to-cell communications or carriers of therapeutics or biomarkers.

**FIGURE 2:**
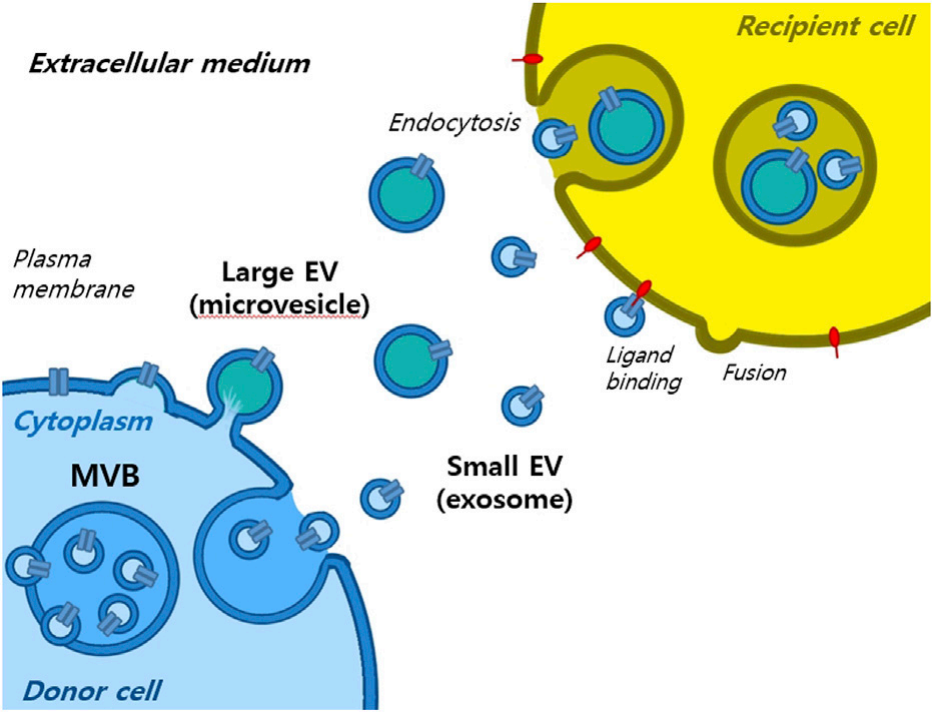
Formation of extracellular vesicles.

Small EVs, formerly referred to as vesicles, have diameters ranging from 40 nm to 200 nm, are released by almost all types of cells (Heldring et al., 2015; Doyle and Wang, 2019; Kim et al., 2021b). The term ‘small EVs’ was applied to vesicles with sizes ranging from 40 to 200 nm formed by fusion of multivesicular endosomes with plasma membranes. However, the 2018 guidelines of the International Society for Extracellular Vesicles proposed the term ‘small extracellular vesicles’, because of isolation difficulties (Théry et al., 2018). Small EVs can be isolated from any bodily fluid, such as plasma, urine, semen, saliva, bronchial fluid, cerebral spinal fluid, breast milk, serum, tears, lymph, bile, or amniotic, synovial, or gastric fluids. Specifically, small EVs are formed by the budding of early endosomes, and during this process, small EVs mature into multivesicular bodies (MVBs) (Russell et al., 2019). Endosomes and MVBs are involved in endocytosis and the trafficking of cellular materials, and especially in protein sorting, recycling, storage, transportation, and release. Eventually, MVBs are transported to lysosomes and are either degraded with their components or fuse with the plasma membrane and release their contents, which include small EVs, into the extracellular space. Small EVs contain nucleic acids, proteins, lipids, cytokines, transcription factor receptors, and other bioactive substances. Exosomal protein components fall into two categories (van der Pol et al., 2012; Akers et al., 2013). The first category includes proteins that participate in vesicle formation and secretion, such as membrane transport and fusion-related proteins (e.g., Rab and GTPases), heat shock proteins (e.g., HSP70 and HSP90), four-transmembrane superfamily proteins (e.g., CD63 and CD81), endosomal sorting complex required transport (ESCRT) complex-related proteins (e.g., Tsg101 and Alix), and integrins. In addition, small EVs contain proteins related to cell-specific progenitors, such as CD45 and MHC-II, derived from antigen-presenting cells, and components related to cell-specific progenitors, such as CD45 and MHC-II. Several studies have shown that small EVs may be involved in physiological and pathological processes and serve as mediators of intercellular communication and material exchange (van der Pol et al., 2012; Akers et al., 2013; Théry et al., 2018; Russell et al., 2019). The formation of small EVs has not been completely elucidated; however, some of the key pathways involved have been explored. Small EVs are formed by the infiltration of cell surface proteins and cell membranes and by the accumulation of bioactive substances in early-sorting endosomes that transform or fuse with late-sorting endosomes under the control of endocytosis-sorting complex and other proteins related to those required to form MVBs. When mature MVBs fuse with the cell membrane, their contents are secreted as EVs (Figure 2).

## 3 Isolation and characterization

### 3.1 Small EVs isolation methods

Small EVs of high purity are required for research and clinical applications, and various methods, such as ultracentrifugation (UC), ultrafiltration (UF), size-exclusion chromatography (SEC), immunoaffinity capture, and polymer precipitation, have been developed for their isolation based on size, shape, density, and surface proteins. However, these methods have their advantages and limitations, and further improvements are required to support the study of small EVs and their applications (Table 1) (Livshits et al., 2015; Popović and de Marco, 2018).

**TABLE 1:** Comparison of exosome isolation methods.

Isolation method	Principle	Potential advantages	Potential disadvantages	Time
Ultracentrifugation (UC)	Sequential centrifugation based on exosome density, size, shape	High accessibility	Time-consuming. might be damaged due to high the speed centrifugation	5–10 h
		High reproducibility	Low purity exosomes	
		Low cost		
Ultrafiltration (UF)	Centrifugation and filtration based on membrane pore	Does not require expensive, special equipment	Time-consuming	3–5 h
		Good portability	Moderate purity of isolated exosomes	
Size exclusion	Separate exosomes from other proteins based on the size difference	High-purity exosomes and good yield	Separating exosomes of similar size is difficult	2–4 h
Chromatography (SEC)			May require ultrafiltration prior SEC	
Immunoaffinity captures	Based on specific interactions between membrane-bound antigens of exosomes and immobilized antibodies	Without the need for specialized equipment	High cost of antibodies	2–6 h
		Highly purity exosomes	Lack of well-defined markers	
			Requirement of skilled personnel	
Polymer precipitation	Altering the solubility or dispersibility of exosomes by use of water-excluding polymers	Easy to use	May be contaminated by proteins and immune complexes	
		0.5–12 h	Without the need for specialized equipment	Require treatment clean-up

#### 3.1.1 Ultracentrifugation

Ultracentrifugation is commonly used to isolate small EVs from biological fluids (Royo et al., 2020) due to its accessibility, reproducibility, and low cost (Gardiner et al., 2016). However, the forces generated can alter small EVs morphology and, consequently, the biological functions of small EVs (Livshits et al., 2015; Popović and de Marco, 2018), and it is difficult to extract small EVs of high purity by UC owing to contamination by soluble and lipid proteins. Furthermore, UC is relatively time-consuming.

#### 3.1.2 Ultrafiltration

Ultrafiltration (UF) involves the use of a membrane with a specified pore diameter range or molecular weight cut-off to isolate particles of a predetermined size (He et al., 2019). Thus, UF is a size-based isolation method used to isolate EVs from cell culture media and separate small EVs and co-vesicles by size. UF can be classified as tandem-configured UF or sequential UF. Tandem-configured UF involves two tandem-configured microfilters with known size-exclusion limits and can separate 20–200 nm and <20 nm particles. Sequential UF is another popular method for small EVs isolation. Extracellular fluids are first passed through a 1,000 nm filter to remove large particles, such as cell debris, cells, and apoptotic bodies, then through a second filter with a 500 kD cut-off to remove free proteins and other small particles, and finally through a 200 nm filter after which small EVs with diameters of 50–200 nm are collected. However, membrane pores are easily blocked. This problem was substantially overcomed by the development of tangential flow filtration (TFF), a form of crossflow filtration, which efficiently minimizes membrane clogging. This technique allows automated recirculation and thus provides high yields. Due to its advantages, TFF-based small EVs preparation has been employed for separating small EVs in various clinical trials.

#### 3.1.3 Size-exclusion chromatography

Size-exclusion chromatography (SEC), also known as gel-filtration chromatography, separates biological samples based on size differences. Macromolecules cannot enter gel pores, whereas small molecules pass through these pores and are eventually eluted. SEC is usually used in combination with UF, which is faster and more productive than UC and does not require expensive equipment. However, membrane pores are easily blocked and separation of similar-size small EVs is difficult (Akbar et al., 2019). On the other hand, combined UF/SEC overcomes the limitations of the two methods and can efficiently separate small EVs from lipoproteins and plasma proteins at extremely high yields (Nordin et al., 2015) and provides small EVs with excellent functionalities.

#### 3.1.4 Immunoaffinity capture

Immunoaffinity capture is an isolation and purification method based on the specific binding of antibodies and ligands to targeted entities in heterogeneous mixtures. Small EVs membranes contain several specific proteins, such as CD63, CD81, CD9, and ESCRT complex-related proteins. Therefore, immunoaffinity capture using EV-related antibodies (CD63, CD81, and CD9) bound to magnetic beads can be used to isolate small EVs selectively. Furthermore, this technique is suitable for separating small EVs with specific origins and for high-throughput studies (Nordin et al., 2015). However, small EVs markers must be optimized because immunoaffinity capture is biased towards separating tetraspanins from EVs. Furthermore, small EVs isolation by immunoaffinity capture can be expensive because of the costs of antibodies and the need for skilled technical personnel.

#### 3.1.5 Polymer precipitation

Polymer precipitation depends primarily on the water affinity of polyethylene glycol (PEG), which sequesters water molecules and facilitates the removal of less soluble components from samples (Witwer et al., 2013; Yang et al., 2020a). PEG reduces the solubility of EVs and enables their separation by low-speed centrifugation or filtration. This precipitation method is straightforward and does not need specialized equipment. However, precipitates may be contaminated by soluble non-exosomal proteins and immune complexes, requiring complicated clean-up steps.

### 3.2 Small EVs characterization

Extracellular vesicles (EVs) are heterogeneous membranous vesicles with different sizes, functions, and origins (Konoshenko et al., 2018), and thus, detailed information on their physicochemical properties (size, shape, density, surface charge, and porosity) is required by those examining their biological features and interactions. Small EVs can be characterized based on their external (morphology and particle size) or internal (membrane proteins, markers, and marker proteins) characteristics (Tiwari et al., 2021).

#### 3.2.1 External characterization

Electron microscopy (EM) is commonly used to determine the small EVs morphologies and sizes (Tiwari et al., 2021), though transmission electron microscopy (TEM), scanning electron microscopy (SEM), cryogenic electron microscopy (cryo-EM), and atomic force microscopy (AFM) have also been used (Tiwari et al., 2021). TEM and SEM can be used to directly observe the internal structures and surfaces of small EVs, respectively. In addition, cryo-EM analyzes small EVs structures in a near-native state, and AFM provides an alternative method for efficiently performing simultaneous nanomechanical and morphological analyses (Raposo et al., 1996; Yuana et al., 2010). Furthermore, nanoparticle tracking analysis can determine particle sizes, concentrations, and distributions and can detect small EVs in the range 40–200 nm, and dynamic light scattering, which uses the same principle as nanoparticle tracking, can also be used to determine EV size distributions.

#### 3.2.2 Internal characterization

Western blotting and ELISA (enzyme-linked immunosorbent analysis) are used to detect the expressions of exosomal marker proteins, though Western blotting is the most commonly used quantitative method. Flow cytometry provides a powerful molecular approach to EV analysis and detects and analyzes laser light scatter at intersections between a flowing liquid and suspended particles using light of specific wavelengths. The technique can also be used to detect small EVs biomarkers (Gardiner et al., 2016) and for the structural and morphological analyses of EVs.

## 4 Small EVs and differences related to adipose tissue-MSC

The transcriptome signatures, biological identities, and functions of mesenchymal stem cells (MSCs) isolated from adult and neonatal tissues, such as bone marrow, adipose, skin, umbilical cord, placenta, and dental tissues, differ significantly depending on their anatomical functions (Banas et al., 2007; Andrukhov et al., 2019; Gan et al., 2020; Levy et al., 2020), which include proliferative and pluripotent differentiation potentials, cellular senescence, secretion, and immunomodulation (Martin et al., 2019). Thus, the regenerative (therapeutic) potentials are much influenced by the origins of MSCs. The ability of MSCs derived from adipose tissue (ADMSCs), the major source of MSCs, to proliferate does not depend significantly on patient age and offers a promising basis for stem cell-based regenerative therapies. ADMSCs can be extracted using a variety of sources and techniques. The main sources are infrapatellar fat pads, epidural fat (EF), lower eyelid fat, and subcutaneous fat (Ye et al., 2016; Hindle et al., 2017), but the techniques and protocols for harvesting and isolating ADMSCs vary by laboratory. ADMSCs have been extensively studied in cartilage tissue engineering, spinal cord injury, and regenerative medicine, but have several drawbacks, as described below.

According to the International Society for Cellular Therapy, MSC phenotypes must meet three criteria; adhesion to culture plates, the expressions of appropriate surface antigens, and multipotent differentiation potential (Dominici et al., 2006; Krampera et al., 2013). Plate adhesion is a characteristic of MSCs (Colter et al., 2000; Jiang et al., 2002) and MSCs exhibit typical morphology and colony formation patterns when observed under a microscope. Immunophenotyping can be performed to confirm the phenotype of MSCs extracted from fat by surface antigen screening and flow cytometry (Al-Saqi et al., 2014), and it has been reported that almost all MSCs are positive (≥95%) for CD73, CD90, and CD105 but negative (≤2%) for CD14 and CD34 (Koellensperger et al., 2014). This negativity for CD14 and CD34 can be used in combination with other criteria to identify MSCs (Sabatini et al., 2005; Wankhade et al., 2016). Furthermore, MSCs can differentiate into osteoblasts, chondrocytes, or adipocytes (Dominici et al., 2006), and differentiation capacities can be confirmed by differentiating the cells into specific lineages using different induction pathways (Pittenger et al., 1999) and subsequent staining, q-PCR (quantitative polymerase chain reaction), and Western blotting.

ADMSCs have therapeutic potential, but their availabilities are limited. Furthermore, the need for strict control over the isolation, collection, storage, and transport of all types of mesenchymal stem cells, including ADMSCs, may limit the efficacy of MSC-based therapies. In addition, the use of living cells presents unavoidable risks of immune rejection and tumor development due to uncontrolled replication. In contrast, small EVs can transmit messenger molecules as biological signals, though the molecules within small EVs may be therapeutic potential or regulating disease environment.

Small EVs are secreted by almost all types of cells and are abundant in body fluids (Colombo et al., 2014; Gheinani et al., 2018). MSC-derived small EVs have functions similar to MSCs that include damage repair, inflammatory response inhibition, and immune system regulation. These small EVs have diameters between 30 and 150 nm and densities of 113–119 g/mL (Théry et al., 2006). It has been reported that MSCs can produce more small EVs than myoblasts, a human acute monocytic leukemia cell line, and a human embryonic kidney cell line (Yeo et al., 2013). However, the action mechanisms of small EVs remain unclear, and the findings of previous studies are controversial. Currently, it is believed that small EVs are more stable than MSCs, have no risk of aneuploidy, and a low probability of immune rejection following allogeneic administration *in vivo*. Thus, they are considered suitable for the treatment of a variety of diseases (Nomura, 2017; Zhang et al., 2019).

However, investigators continue to determine whether small EVs extracted from ADMSCs have organ-dependent effects. Small EVs extracted from infrapatellar fat pads, EF, and subcutaneous fat-derived MSCs have homogenous diameters between 40 and 200 nm in size. In a previous study, nanoparticle tracking analysis showed that small EVs in sediment had diameters of 40–200 nm (Nomura, 2017). The levels of the small EVs-specific surface proteins CD9 and TSG101 were confirmed by Western blotting, but results showed an increase in protein levels. Notably, high-quality small EVs produced by EF-derived MSCs extracted using TFF did not express GAPDH (Nomura, 2017).

Small EVs isolated from EF-derived MSCs have been effective at restoring spinal function by reducing inflammatory response (Kim et al., 2021a; Kim et al., 2021b; Sung et al., 2022a). In addition, neuronal cell marker expressions are elevated in EF-derived MSCs. These results indicate that the characteristics of adipose tissue are closely related to those of adjacent organs and suggest that small EVs-based treatment is related to nerve recovery and eminently suitable for experimental purposes (Kruglikov and Scherer, 2016; Solmaz et al., 2020; Sung et al., 2022b).

## 5 Therapeutic potential of small EVs for improving neural structure

The treatment of peripheral nerve and spinal cord injuries is extremely challenging (Kim et al., 2020; Tsuji et al., 2021; Sung et al., 2022b). Treatment methods implemented to date are suboptimal, and no protocol is available for directly treating nerves. When nerves are damaged, inflammatory cells infiltrate to form fibrous glial scars and limit axonal regeneration (Silver and Miller, 2004; Donnelly and Popovich, 2008; Shao et al., 2019). Recently characterized MSCs have emerged as a new treatment approach (Silver and Miller, 2004; Vismara et al., 2017; Shao et al., 2019), but the use of MSC transplantation is limited by the risks of immune response and tumorigenesis induced by uncontrolled cell growth. However, according to a recent study, small EVs improve nerve regeneration and are free of the risks associated with living cells (Zhang et al., 2017). In the nervous system, small EVs play an important role in the regulation of regeneration, and miRNAs in small EVs derived from Schwann cells, macrophages, and MSCs promote peripheral nerve regeneration (Pegtel et al., 2014). Small EVs consist of constitutive and cargo molecules, the latter of which include proteins (tetraspanins, TSG101, and heat shock proteins), lipids, and genetic materials (DNA, mRNA, miRNA, and rRNA) (Qing et al., 2018a). In previous studies, through proteomic analysis, histone deacetylases (HDACs), amyloid-beta A4 protein (APP), and integrin beta-1 (ITGB1) among several exosomal proteins secreted by adipose tissue-derived stem cells are highly involved in a process of nerve regeneration (Qing et al., 2018a; Toh et al., 2018; Rau et al., 2021). Meanwhile, some studies have pointed out that the specific miRNAs of small EVs related to the regeneration processes may not be present sufficiently (Toh et al., 2018; Albanese et al., 2021). However, most studies about EVs have shown that small EVs can play a critical role for regeneration and inflammation regulation, by a diverse of mechanisms (such as messenger delivery, communication between specific cells, and paracrine pathway) (Santonocito et al., 2014; Sun et al., 2018; Yue et al., 2020). Baglio et al. demonstrated tissue-specific microenvironments affect small EVs miRNA contents and that small EVs miRNAs affect nerve regeneration through intercellular signaling (Baglio et al., 2015).

**FIGURE 3:**
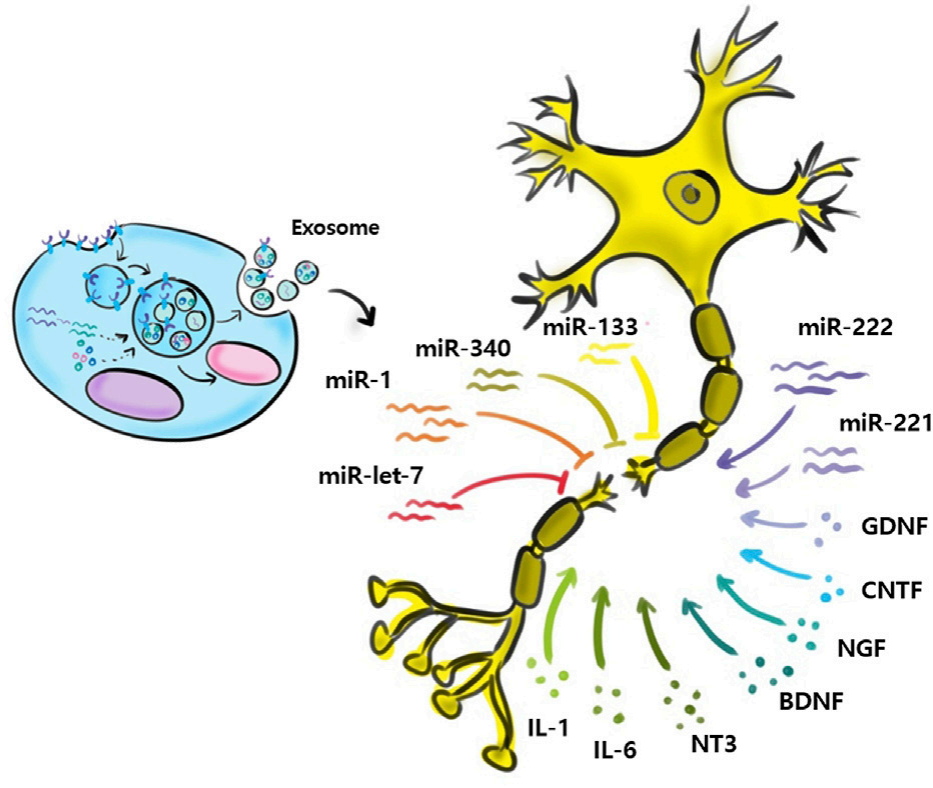
Exosomal miRNAs and nerve regeneration.

Relatively few studies have addressed small EVs contributions to nerve regeneration (Figure 3). Studies performed to date have shown M2 macrophages, MSCs, Schwann cells, microglial, neurons, sensory neurons, motor neurons, and astrocytes secrete small EVs, and that small EVs also contain various miRNAs (Qing et al., 2018b; Zhang et al., 2018; Silvestro and Mazzon, 2022). Zhang et al. (Santonocito et al., 2014) reported that migration and proliferation were promoted when small EVs containing miR-223 were secreted by M2 macrophages co-cultured with Schwann cells and that Schwann cell migration and proliferation were reduced when they were incubated with miR-223-inhibited M2 macrophages. In addition, M2 macrophage-derived small EVs were found to induce axon growth by increasing nerve growth factor and laminin expressions (Zhan et al., 2015). Small EVs secreted by MSCs contain the miRNA-17-92 cluster, miRNA-133b, and miRNA let-7b and play essential regulatory roles during axonal growth and inflammation through various signaling pathways. The miRNA-17-92 cluster promotes axonal growth by activating the PTEN/mTOR signaling pathway (Zhang et al., 2017). miRNA-133b mediates the RhoA and ERK 1/2/CREB signaling pathways to improve neural plasticity and functional recovery, whereas miRNA-133b exhibits anti-apoptotic effects. Shen et al. (2018) suggested miRNA-133b as a potential therapeutic target for treating brain injuries and reported miRNA-340 secretion by Schwann cells helps remove debris from damaged neurons and promotes axonal regrowth. Most studies on miRNA-containing small EVs have focused on the effects of miRNAs on nerve regeneration. However, Li et al. (2017) confirmed that miRNAs participate in the removal of debris that interferes with nerve regeneration. miRNA-124-3p in small EVs, secreted by microglial cells after brain injury, induced the differentiation of M2 macrophages, exhibited anti-inflammatory effects, and suppressed neuroinflammation by inhibiting the mTOR signaling pathway (Huang et al., 2018). Small EVs secreted by damaged neurons reportedly contain miRNA-212, miRNA-21, miRNA-146, miRNA-7a, and miRNA-7b. In a study by Harrison et al., miRNA-212 expression was diminished in neurons of a damaged brain and the expressions of miRNA-21, miRNA-146, miRNA-7a, and miRNA-7b were enhanced. miRNA-21 expression was elevated mainly near lesions in damaged neurons and led to the activation of microglia (Harrison et al., 2016). Specific miRNAs associated with nerve regeneration are summarized in Table 2. (Verrier et al., 2009; Strickland et al., 2011; Viader et al., 2011; Liu et al., 2013; Wu and Murashov, 2013; Zhou et al., 2015; Zou et al., 2015; Yi et al., 2016; Zhou et al., 2017; Dai et al., 2018; Cui et al., 2019; Li et al., 2019; Lv et al., 2019; Yu et al., 2019).

**TABLE 2:** miRNAs that regulate nerve regeneration.

Functions	miRNAs	Target points	References
Axonal extension enhancement	miR-21	SPRY2	Strickland et al. (2011)
	miR-26a/miR-29a	PTEN	Zou et al. (2015)
	miR-431	Kremen 1	Wu and Murashov (2013)
Apoptosis inhibition	miR-21/miR-222	TIMP-3	Zhou et al. (2015)
	miR-138	SIRT1	Liu et al. (2013)
	miR-448	Bcl2	Lv et al. (2019)
Schwann cell proliferation	miR-34a	NOTCH1/Ccnd1	Viader et al. (2011)
	miR-sc3	Astn1	Yi et al. (2016)
	miR-29a	PMP22	Verrier et al. (2009)
Neuroregeneration/proliferation	miR-124	PDXK	Cui et al. (2019)
	miR-29b	NF-200/GAP-43	Yu et al. (2019)
	miR-125b	JAK1/STAT1	Dai et al. (2018)
	miR-372	KIF3B/NOSIP	Li et al. (2019)
	miR-210	PTP1B	Zhou et al. (2017)

Small EVs have potential therapeutic applications because of their endogenous properties. However, bioengineering improvements are needed to address identified clinical and commercial limitations. The clinical applications of small EVs for immune modulation, tissue regeneration and repair, and combination therapy are being investigated. In addition, small EVs can be used for drug delivery (Sluijter et al., 2018; Pirisinu et al., 2022) or as paracrine signaling components in stem cell- or progenitor cell-based therapies. The ability of small EVs to deliver biological and pharmaceutical molecules to specific tissues and cells has generated considerable interest among those developing biocompatible drug delivery systems. At present, 224 studies on small EVs are registered at https://www.clinicaltrials.gov. The majority are clinical trials aimed at developing preclinical therapeutics for wound healing, whereas others address diagnostic and biomarker research topics.

Research interest in small EVs-based therapeutics has increased, and thus, optimizations, improvements, and standardizations of designs, production processes, and clinical administration methods have moved to the fore. In particular, several fundamental challenges, such as purity, production yields, standardization of small EVs generation, and the determination and quantification of potency and molecular activity, remain. Consequently, several studies have been conducted to address these challenges (Lener et al., 2015; Reiner et al., 2017; Gandham et al., 2020; Rankin-Turner et al., 2021).

## 6 Current issues and future perspectives

### 6.1 Cell culture conditions and small EVs isolation

The cell culture conditions used to produce small EVs vary among laboratories (Ludwig et al., 2019), and small EVs yields and cargoes are highly dependent on the conditions used to culture parent cells. Cellular environments such as culture containers, cell numbers, medium compositions, and hypoxia as well as cell type have significant effects (Jiang et al., 2022), and researchers find it difficult to identify suitable conditions that enable the effective isolation of required small EVs. Although recently, a method was devised to isolate small EVs at high purity (Karimi et al., 2022). Non-etheless, efforts are required to devise robust, reproducible methods and define standards for the small EVs produced. Small EVs separation methods should be reproducible and accessible and produce high yields of high-purity small EVs at a low cost. In addition, standardization of the protocols used by laboratories is essential (Liu et al., 2022).

Most scientific studies on small EVs require only small numbers because they are performed at the laboratory scale. Thus, to develop small EVs-based treatments, clinical trials that focus on small EVs stability, reproducibility, and convenience are required (Soekmadji et al., 2018). Furthermore, research is needed to establish standards for all aspects of the processes used, including cell culture conditions and separation and purification methods. To address these issues, the international societies of EVs (SOCRATES, ISEV, ISCT, and ISBT) have proposed the specific criteria for MSC-EVs to facilitate data sharing and comparison, helping to advance small-EVs into clinical applications. (Witwer et al., 2019; Gimona et al., 2021).

### 6.2 Scalability and standardization of small EVs production for clinical use

The clinical use of small EVs requires that standard, scalable, and cost-effective production systems be developed (Lener et al., 2015). Small EVs-based therapeutics require large amounts of small EVs of high purity that meet treatment efficacy requirements (Whitford and Guterstam, 2019). To address the limitations of protocols devised for clinical-grade large-scale production, efforts are required to improve understanding and standardize variables that affect small EVs production.

Small EVs isolation can be improved by careful consideration of variables, such as the suitabilities of donor cells (epithelial and mesenchymal cells) and their growth statuses. Fat-derived small EVs are suitable therapeutic candidates and have advantages in the regenerative medicine field in terms of wound healing and tissue restoration, as well as their anti-inflammatory properties and low immunogenicities (Tan et al., 2018; Shukla et al., 2020). Cell culture systems, media, and supplements influence the culture of fat-derived cells, and their optimizations can improve small EVs yields (Patel et al., 2017; Palviainen et al., 2019; Shukla et al., 2020; Wang et al., 2020; Zhu et al., 2021). However, the extents to which these optimizations affect small EVs composition, efficacy, and other factors related to therapeutic use have yet to be determined (Colao et al., 2018; Adlerz et al., 2020).

The development of a reliable potency assay for MSC-EVs is challenging due to their complex and diverse characteristics, which make it difficult to define potency metrics. Mario et al. proposed the use of a matrix array of multiple assays that measure various attributes of MSC-EVs that correlate with their intended use, as guidance from regulatory agencies like the European Medicines Evaluation Agency, ICH, and US FDA can help with this development (Gimona et al., 2021). Additionally, developing quality control measures and reproducible manufacturing processes for MSC-EVs is challenging due to the wide variability in MSC-EV preparations caused by the lack of standardized quality assurance and functional assays. To overcome this challenge, Kenneth et al. suggest developing minimal quantifiable metrics to harmonize the definition of MSC-EVs and provide a denominator for comparative manufacturing and functional testing. Kenneth et al. recommended identifying metrics such as the ratio of MSC to non-MSC surface antigens, ratio of membrane lipids to protein, ratio of specific lipids, concentration of membrane lipid vesicles, vesicle integrity, and biological activity and validating each metric by comparing them to a well-characterized MSC-EV preparation (Witwer et al., 2019). Collaboration among researchers is also crucial for promoting MSC-EV research and applications.

The clinical application of small EVs also requires judicious consideration of the characteristics of mother cells, such as their therapeutic and anti-inflammatory effects. Immortalization of mother cells by transfection or genetic engineering requires thorough risk analysis of cells, including transformed cells, and derived small EVs. For therapeutic small EVs, the immortalization of mother cells enables the sustained production of small EVs without affecting therapeutic efficacies or immunosuppressive activities. However, the immortalization of mother cells has been associated with stability issues (Chen et al., 2011; Zhou and Kalluri, 2020; Herrmann et al., 2021). Therapeutic small EVs are being increasingly produced in parallel with understanding of the biological functions of MSCs (Cha et al., 2018). Several studies have described the morphologies, structural characteristics, and expressions of cytokines and miRNAs in small EVs isolated from cells and *in vitro* quality control considerations (Tan et al., 2018; Shukla et al., 2020).

Large-scale commercial production is an essential requirement of small EVs-based therapeutics being considered for clinical applications. Cells can be cultured in large-capacity stirred tanks or platform rocker wave bags, whereas small-scale cultures can be performed in multilayer flasks, spinners, wave bags, or fixed-bed reactors (Whitford and Guterstam, 2019). Currently, large-scale production of therapeutic small EVs is conducted by filtration (0.2 μm), UC, or size-based chromatographic fractionation. Good manufacturing practice (GMP)-grade small EVs have been isolated using TFF from human progenitor cells for small EVs-based therapy (Andriolo et al., 2018). The production of sustainable small EVs is essential for successful applications and developments of small EVs-based therapeutics. In 2020, cellular nanopores were reported to increase the production of small EVs as nucleic acid carriers requiring transcriptional manipulation (Yang et al., 2020b). Careful screening of mother cells with specific therapeutic and bioactive effects is essential for long-term cell growth. Studies on the abilities of small EVs to encapsulate drugs are in their infancy, and encapsulation efficiencies must be improved.

Furthermore, stability, preservation, and storage are also key requirements of small EVs-based therapeutics (Jeyaram and Jay, 2018). Stock storage conditions have considerable impacts on small EVs functions and therapeutic applications. Several studies have evaluated small EVs stability at different storage temperatures and after freeze-thaw cycles, and short- and long-term stock storage temperatures of −20°C and −80°C, respectively, have been recommended (Wu et al., 2021).

Overall, the transition to clinical development requires industry-level scale-up and understanding of the effects of variables during the early stage of development. Production systems must produce small EVs consistently at high yields and purities. Non-etheless, despite current obstacles, we are confident that the clinical and commercial requirements of therapeutic small EVs-based production can be fully met by modifying existing techniques.

## 7 Conclusion

The treatment of neural injury is an unresolved challenge, and currently, no therapeutic modality can effectively restore lost functions. Utilization of the various direct and indirect pathways associated with the regeneration of injured tissues by small EVs offers promise for the improved management of neural injuries. However, issues related to small EVs, such as tissue source, isolation, purification, and amplification, must be comprehensively addressed. Rapid, inexpensive, straightforward, standardized isolation and purification procedures are required to generate small EVs at high yields and purities with intact biological activities. In addition, trials are needed to establish the clinical effectiveness and safety of small EVs in humans. Furthermore, studies should be conducted to establish a comprehensive theoretical basis for the clinical application of small EVs and to provide directions for the treatment of neural injuries.
