# Supplementary material for: Therapeutic potential of small extracellular vesicles derived from mesenchymal stem cells for spinal cord and nerve injury
Source: Front Cell Dev Biol. 2023 Mar 22;11:1151357. doi: 10.3389/fcell.2023.1151357 (PMC10073723; doi:10.3389/fcell.2023.1151357)
Supplement: Supplementary file 1 [file Table1.DOCX]

Table 1. Comparison of exosome isolation methods

| **Isolation method** | **Principle** | **Potential advantages** | **Potential disadvantages** | **Time** |
| --- | --- | --- | --- | --- |
| Ultracentrifugation (UC) | Sequential centrifugation based on exosome density, size, shape | High accessibility  High reproducibility  Low cost | Time-consuming.  might be damaged due to high the speed centrifugation  Low purity exosomes | 5-10 hrs |
| Ultrafiltration  (UF) | Centrifugation and filtration based on membrane pore | Does not require expensive, special equipment  Good portability | Time-consuming  Moderate purity of isolated exosomes | 3-5 hrs |
| Size exclusion  Chromatography (SEC) | Separate exosomes from other proteins based on the size difference | High-purity exosomes and good yield | Separating exosomes of similar size is difficult  May require ultrafiltration prior SEC | 2-4 hrs |
| Immunoaffinity captures | Based on specific interactions between membrane-bound antigens of exosomes and immobilized antibodies | Without the need for specialized equipment  Highly purity exosomes | High cost of antibodies  Lack of well-defined markers  Requirement of skilled personnel | 2-6 hrs |
| Polymer precipitation | Altering the solubility or dispersibility of exosomes by use of water-excluding polymers | Easy to use  Without the need for specialized equipment | May be contaminated by proteins and immune complexes  Require treatment clean-up | 0.5-12 hrs |
