# Supplementary material for: Therapeutic potential of small extracellular vesicles derived from mesenchymal stem cells for spinal cord and nerve injury
Source: Front Cell Dev Biol. 2023 Mar 22;11:1151357. doi: 10.3389/fcell.2023.1151357 (PMC10073723; doi:10.3389/fcell.2023.1151357)
Supplement: Supplementary file 2 [file Table2.DOCX]

Table 2. miRNAs that regulate nerve regeneration

| Functions | miRNAs | Target points | References |
| --- | --- | --- | --- |
| Axonal extension enhancement | miR-21 | SPRY2 | [90] |
|  | miR-26a / miR-29a | PTEN | [91] |
|  | miR-431 | Kremen 1 | [92] |
| Apoptosis inhibition | miR-21 / miR-222 | TIMP-3 | [93] |
|  | miR-138 | SIRT1 | [94] |
|  | miR-448 | Bcl2 | [95] |
| Schwann cell proliferation | miR-34a | NOTCH1 / Ccnd1 | [96] |
|  | miR-sc3 | Astn1 | [99] |
|  | miR-29a | PMP22 | [98] |
| Neuroregeneration/ proliferation | miR-124 | PDXK | [97] |
|  | miR-29b | NF-200 / GAP-43 | [100] |
|  | miR-125b | JAK1 / STAT1 | [101] |
|  | miR-372 | KIF3B / NOSIP | [102] |
|  | miR-210 | PTP1B | [103] |
